# Selection of suitable reference genes for assessing gene expression in pearl millet under different abiotic stresses and their combinations

**DOI:** 10.1038/srep23036

**Published:** 2016-03-14

**Authors:** Radha Shivhare, Charu Lata

**Affiliations:** 1 CSIR-National Botanical Research Institute, Rana Pratap Marg, Lucknow-226001, India; 2 National Research Centre on Plant Biotechnology, Pusa Campus, New Delhi-110012, India

## Abstract

Pearl millet [*Pennisetum glaucum* (L.) R. Br.] a widely used grain and forage crop, is grown in areas frequented with one or more abiotic stresses, has superior drought and heat tolerance and considered a model crop for stress tolerance studies. Selection of suitable reference genes for quantification of target stress-responsive gene expression through quantitative real-time (qRT)-PCR is important for elucidating the molecular mechanisms of improved stress tolerance. For precise normalization of gene expression data in pearl millet, ten candidate reference genes were examined in various developmental tissues as well as under different individual abiotic stresses and their combinations at 1 h (early) and 24 h (late) of stress using geNorm, NormFinder and RefFinder algorithms. Our results revealed *EF-1α* and *UBC-E2* as the best reference genes across all samples, the specificity of which was confirmed by assessing the relative expression of a *PgAP2 like-ERF* gene that suggested use of these two reference genes is sufficient for accurate transcript normalization under different stress conditions. To our knowledge this is the first report on validation of reference genes under different individual and multiple abiotic stresses in pearl millet. The study can further facilitate fastidious discovery of stress-tolerance genes in this important stress-tolerant crop.

Plants being sessile in nature are forced to thrive in adverse environmental conditions. Drought, salinity and temperature extremes (heat and cold) are major abiotic stresses that challenge production and productivity of crop plants^1^,^2^. The adverse effects of these stresses on crop plants are further compounded due to changing climate worldwide^3^. Further it has also been reported that levels of abscisic acid (ABA), a plant growth regulator, also increases under abiotic stresses^4^,^5^. Though individual stress conditions such as drought, salinity or heat have been the focus of intense research, not much study has been carried out on the combination of different abiotic stresses such as drought and heat, drought and salinity, salinity and heat etc. which frequently affect growth and yield potential of crops and other plants in field conditions across the globe^6^,^7^. There have been quite a few reports suggesting that response of plants to two or more different abiotic stresses is unique and quite distinct from the response of those exposed to individual stress^8^. Generally, more than one stress factors occurring simultaneously, interact with each other in additive manner and trigger more damage than when present individually^9^,^10^. Therefore it is necessary to focus on development of crops varieties that can endure multiple environmental stress factors. Since crop plants are subjected to one or more abiotic stresses concurrently under field conditions, it is thus necessary to make conscious efforts towards mimicking these conditions in laboratory as well^7^,^9^,^11^. Further an overlap in the expression patterns of stress responsive genes after drought, salt, heat, cold, or ABA treatments or after combinations of stresses has also been reported^8^,^12^. Subsequently several studies have been taken up recently to monitor the effects of abiotic stress combinations on crop plants as well as to elucidate the molecular mechanism(s) of stress tolerance^8^,^12^,^13^,^14^,^15^.

Pearl millet [*Pennisetum glaucum* (L.) R. Br.] is an important small-grained C_4_ panicoid crop grown for forage, grain and stover in the arid and semi-arid regions of Asia and Africa^16^. It is the sixth most important cereal crops after rice, wheat, maize, barley and sorghum with excellent nutrient composition^17^ and is also considered a potential biofuel grain feedstock^2^,^18^; http://ag.fvsu.edu/index.php/research/bioenergy/). It usually thrive in areas with scanty rainfall and is also well adapted to various abiotic stresses such as drought, high temperature, salinity etc. whether occurring individually or in combination making it an ideal crop for functional genomic studies to understand the molecular basis of abiotic stress tolerance and adaptation^2^,^16^,^19^. Only a limited amount of genome sequence information is available in pearl millet that greatly hinders gene discovery, functional validation, expression profiling, and ultimately crop improvement programs. Quantification of variable transcriptomes and analysing differential expression of stress responsive genes in diverse biological samples and experimental conditions are important functional genomic approaches to investigate the molecular basis of stress tolerance involving complex regulatory gene networks^20^. In this regard, qRT-PCR is a widely used technique to quantify transcriptional abundance of numerous individual genes owing to its high sensitivity, specificity and synchronized quantification of gene expression in diverse samples with a broad quantification range of up to several orders of magnitude in comparison to conventional techniques such as reverse transcription (RT)-PCR or northern hybridization^20^. However relative quantification of gene expression using qRT-PCR is highly influenced by the expression stability of internal control or reference genes used for transcript normalization of target genes. The use of inappropriate or unstable reference gene(s) can seriously impact the transcript quantification results leading to false inferences or misinterpretations^21^,^22^. Accurate normalization is thus necessary for obtaining biologically meaningful expression data and hence qRT-PCR analysis greatly depends upon careful selection of reliable reference gene(s) which should be stably expressed across various tissue samples, developmental stages and experimental conditions^23^,^24^. Numerous studies have been carried out until now in various economically important cereal crops for determining the stability of reference genes in various developmental stages and abiotic stress conditions^25^,^26^,^27^. However to our knowledge no systematic study has been carried out in any cereal crop for validation of reference genes under different individual abiotic stresses and their various combinations at different durations of stress which are a must for the normalization of gene transcripts that are expressed and regulated under multiple stress conditions. Hence considering the inevitability of crop- as well as stress-specific internal control genes for accurate normalization of transcripts in qRT-PCR, the present study was carried out to evaluate the expression stability of 10 candidate reference genes in pearl millet subjected to individual or combination of abiotic stresses and or abscisic acid (ABA), a stress hormone at early (1h) and late (24h) stress durations as well as in different developmental tissues of two contrasting genotypes.

## Results

### Selection of candidate reference genes, sample size, primer specificity and amplification efficiency

A total of ten potential candidate reference genes including both traditional housekeeping as well as new reference genes namely, *actin (ACT)*, *elongation factor 1α (EF-1α)*, *eukaryotic initiation factor 4A (eIF4A)*, *glutaredoxin (GlutR)*, *heat shock protein 90 (HSP90)*, *malate dehydrogenase (MDH)*, *protein phosphatase 2A (PP2A)*, *ribosomal protein L20 (RPL20)*, *tonoplast intrinsic protein 41 (TIP41)* and *ubiquitin-conjugating enzyme E2 (UBC-E2)* were identified for gene expression studies using qRT-PCR in pearl millet. Out of 10 reference genes sequences of 8 were retrieved from pearl millet and those of *PP2A* and *TIP41*were obtained from foxtail millet.

The quantification of cycling threshold (Ct) or quantitative cycle (Cq) values through qRT-PCR and calculation of coefficient of variation (CV) for 10 candidate reference genes was carried out in an experimental set of a total of 204 samples (n = 204) from two contrasting pearl millet genotypes namely PRLT2/89-33 and H77/833-2, which are also parents of a mapping population, subjected to different individual abiotic stresses or ABA or combinations of individual stresses and ABA or in developmental tissues (Table 1). The individual abiotic stress experimental set comprised a total of 60 samples from 4 stresses namely, drought, salt, heat and cold, and from a hormone stimulus ABA at 1 h and 24 h of stress, whereas multiple stresses set included 96 samples from 8 treatments i.e. drought + salt, drought + heat, drought + ABA, salt + ABA, heat + ABA, cold + ABA, drought + salt + ABA, and drought + salt + heat + ABA from both genotypes. Both individual and multiple stresses included one control sample of each genotype without stress (n = 6). The experimental set of developmental tissues consisted of 42 samples including leaf, root, stem, stem sheath, panicle at booting stage, panicle at grain filling stage and seeds of both genotypes.

The specificity and accuracy of each qRT-PCR primer pair was determined through agarose gel electrophoresis and melt curve analysis for each gene (Supplementary Fig. S1) where single amplicon or single peak, respectively suggested the absence of primer dimmers or non-specific PCR products. The PCR amplification efficiency (*E*) is dependent on the assay, master mix performance, and sample quality. The amplification efficiencies and correlation coefficients (*R*^*2*^) of the ten candidate reference genes were determined using the slopes of the standard curves obtained by serial dilutions which fall in the acceptable range of 90–100% for *E*, and 0.80–1.00 for the *R*^2^. The description of 10 candidate reference genes, accession numbers, primer sequences, amplicon lengths, and amplification efficiency are enlisted in Supplementary Table S1.

### Expression profiling of candidate reference genes

**Figure 1:**
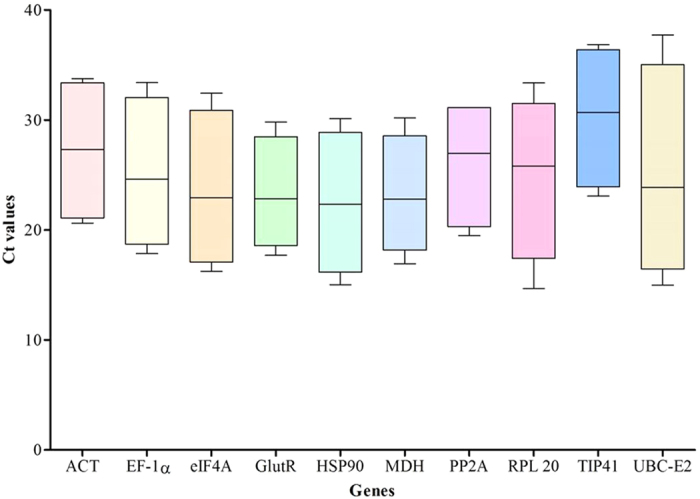
Ct values of candidate reference genes across all samples. Line across the box depict the median value and inside the box show the Ct values. The top and bottom whiskers are determine by the 5^th^ and 95^th^ percentiles, respectively.

Expression levels of all 10 candidate reference genes were determined by qRT-PCR and the Ct values showed differential transcript levels in the samples examined with lower Ct values suggesting higher transcript abundance and vice versa. The Ct values were monitored under four groups including all samples, individual abiotic stresses, multiple abiotic stresses, and developmental tissue samples. The mean Ct values of 10 potential reference genes ranged from 15.5 to 34.7 (Table 2; Fig. 1). The distribution of Ct values is listed in Supplementary Tables S2, S3 and S4. In the all samples set, the mean Ct values showed a minimum of 21.7 ± 3.4 and maximum of 28.9 ± 2.5 for highest and lowest expression levels for *RPL20* and *TIP41*, respectively. *RPL20* and *TIP41* also showed minimum and maximum average Ct values, respectively in individual stress sample set as well as in multiple stress samples set. On the other hand in the developmental tissue set, *UBC-E2* has minimum average Ct value of 21.1 ± 4.8 while *TIP41* has maximum average Ct value of 29.4 ± 3.7. Further CV of the Ct values was also calculated to evaluate the expression levels of candidate reference genes under all four experimental sets, where lower values represent lower variability or maximum stability. The CV of 10 reference genes among all samples ranged between 4 to 15%. *EF-1α* was the least variable reference gene with a CV of 4.5% among 10 candidate reference genes studied and *MDH* was the most variable with a CV of 15.7%. The stability ranking of all candidate reference genes on the basis of CV values is as follows: *EF-1α* < *PP2A* < *GlutR* < *UBC-E2* < *ACT* < *RPL20* < *eIF4A* < *TIP41* < *HSP 90* < *MDH* (Table 1).

### Stability ranking of the candidate reference genes

Expression stability of the candidate reference genes were determined by geNorm, NormFinder and RefFinder which evaluated the stability ranking of each reference gene using the Ct values across all the experimental sets and tissue samples (Tables 3, 4, 5, 6). The stability ranking analyses done by each of the three softwares is detailed in the following sections:

### geNorm analysis

**Figure 2:**
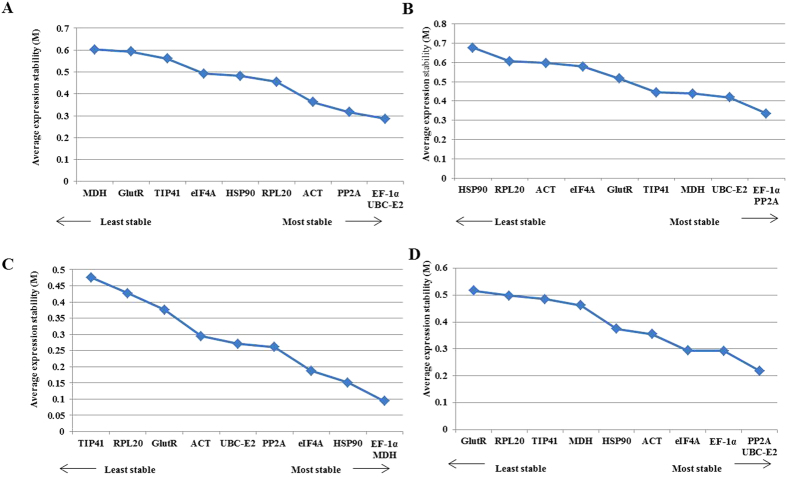
Expression stability and ranking of 10 candidate reference genes as calculated by geNorm in the individual stress samples set (**A**), multiple stress samples set (**B**), developmental tissue samples set (**C**) and all samples set (**D**). A lower value of average expression stability (M) indicates most stable expression.

The gene expression stability of all the 10 candidate reference genes was evaluated using geNorm statistical algorithm. This software determines the normalization value based on the geometric mean of various candidate reference genes and mean pair wise variation of each gene from all the reference genes in a given set of samples. According to geNorm analysis the cut-off range of stability value (M) is <1.5, so the gene with lowest M value is considered to be the most stable reference gene in terms of gene expression and vice versa. We analyzed our data for all four experimental sets and found that all the 10 candidate reference genes exhibited high expression stability with low (<0.7) M values which were much below the default limit of 1.5. Among all samples, *PP2A* and *UBC-E2* have the least M value of 0.22 followed by *EF-1α* and *eIF4A* with M value of 0.29, however *GlutR* exhibited highest M value of 0.52 indicating that *PP2A* and *UBC-E2* were most stable in expression and *GlutR* the least. On the other hand in the individual and multiple stress sample sets, *EF-1α*/*UBC-E*2 and *Ef-1α*/*PP2A* had the lowest M values of 0.28 and 0.33, respectively among all 10 reference genes studied suggesting their higher expression stability, whereas *MDH* (0.60) and *RPL20* (0.68), respectively were referred as least stable genes for normalization in both these sample sets. In developmental tissues experimental set, *EF-1α* and *MDH* with M value 0.094 were ranked as most stable reference genes while *TIP41* (0.48) was ranked least stable. Our results thus suggested *EF-1α* to be the most stable candidate reference gene in three out of four experimental sets (Supplementary Table S5, Fig. 2).

**Figure 3:**
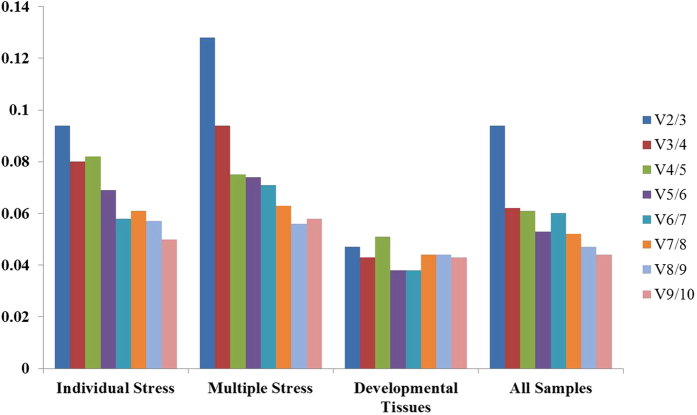
Pairwise variation (V) analysis to determine the optimal number of control genes for accurate transcript normalization in all the four experimental sets. geNorm calculates pairwise variation (Vn/Vn+1) for the normalization factors NFn and NF+1 to determine (V<0.15) the optimal number of reference genes.

geNorm algorithm was also used to determine the optimal number of reference genes as inclusion of two or more internal control genes for transcript normalization in qRT-PCR experiments is considered better for obtaining precise and steady results. geNorm determines the pairwise variation (V_n_/V_n+1_) between sequential normalization factors NF (NF_n_ and NF_n+1_) in each sample set^28^. geNorm first calculates the NF for two highly expressed reference genes and then for remaining genes by adding each gene one by one in order of their decreasing expression stability^20^,^25^,^28^,^29^. For the entire four sets, all pairwise variations were below the cut-off value of 0.15 (Fig. 3).

### NormFinder analysis

Expression level of 10 candidate reference genes were also evaluated using NormFinder which is an excel based mathematical tool that analyzes each sample set individually and also estimates intra- and inter-group variation in expression across different sample sets. NormFinder ranks the control genes on the basis of their stability value (SV) where lower stability value represents higher gene expression stability and vice versa. For the all samples set *EF-1α*, *PP2A* and *UBC-E2* were identified as the best three optimal internal control genes with SV of 0.061, 0.064 and 0.089 while *RPL20* with SV 0.148 exhibited utmost variation. *UBC-E2* (0.048) and *PP2A* (0.103) were the top two reference genes in the individual stress sample set, and *PP2A* (0.097) and *EF-1α* (0.159) topped the multiple stress samples set. While *MDH* (0.032) and *HSP90* (0.034) were ranked the most stable genes in the developmental tissue samples set (Supplementary Table S6).

### RefFinder analysis

Stability ranking of 10 candidate reference genes across four experimental sets was further confirmed using comprehensive ranking method by RefFinder. It is an online available web tool that compares data generated from different software’s (geNorm, NormFinder, SI, ΔCt and BestKeeper) and gives comprehensive ranking to confirm the stability ranking via different programs. In this study the data generated by geNorm and NormFinder across all four sets were compared by RefFinder. *EF-1α* with Geomean ranking values of 1.00 and 1.19 for all and individual as well as multiple stress samples set, was ranked the most stable, and hence best reference gene for accurate transcript normalization except the developmental tissue set where *MDH* was given the highest Geomean ranking value of 1.57 (Supplementary Table S7).

### Expression analysis of an *AP2-like ERF* gene from pearl millet under individual and multiple abiotic stresses for reference genes validation

**Figure 4:**
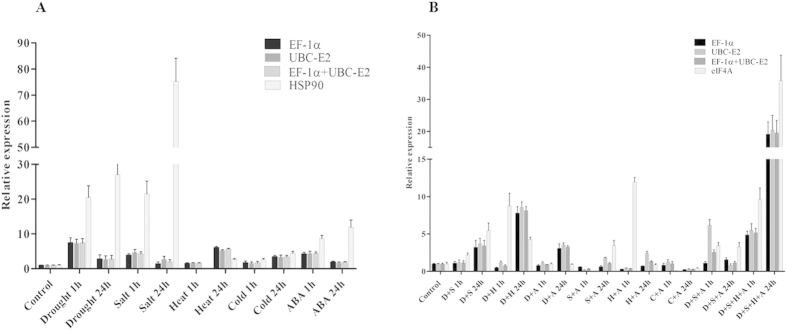
Relative expression of *PgAP2 like-ERF* using selected reference genes including the most and the least stable reference genes for transcript normalization following (**A**) individual, and (**B**) multiple stress treatments after 0, 1 and 24 h. Bar indicate the standard error (±SE) evaluated from three biological replicates.

In order to validate the selection of most and least stable reference genes from this study, the relative expression patterns of an objective gene namely *PgAP2-like ERF*, a transcription factor found to be up-regulated (>2.0 fold) during transcriptome analysis of PRLT2/89-33 and H77/833-2 subjected to drought stress (data from another study; not published), was analyzed under individual and combinations of abiotic stresses at 1 h and 24 h in the tolerant genotype PRLT2/89-33 (Fig. 4). For this overall ranked two most stable (*EF-1α* and *UBC-E2*) reference genes for both single and multiple abiotic stresses, and two least stable reference genes namely, *HSP90* for individual abiotic stresses, and *eIF4A* for multiple abiotic stresses as deduced by RefFinder, were used for the validation (Tables 3 and 4). Relative transcript accumulation of *PgAP2-like ERF* under individual or multiple abiotic stresses was found to be unbiased when *EF-1α*, *UBC-E2* or combination of *EF-1α* and *UBC-E2* were used confirming high level of expression stability of these two genes. Using *EF-1α*, *UBC-E2* or *EF-1α* + *UBC-E2*, the expression of *PgAP2-like ERF* in leaves of the tolerant pearl millet genotype increased under drought, salinity and ABA at 1 h and 24 h with strong expression at 1 h of stress, while the gene was found to be up-regulated under heat and cold stress at 24 h (Fig. 4A). However *PgAP2-like ERF* gene showed relative up-regulation at late time point (24 h) for all stress combinations when *EF-1α*, *UBC-E2* or *EF-1α* + *UBC-E2* were used for transcript normalization (Fig. 4B). The relative expression pattern of *AP2-like ERF* showed strong fluctuations and failed to achieve consistency when *HSP90* and *eIF4A* was used for transcript normalization under individual and multiple abiotic stresses, respectively.

## Discussion

Abiotic stress conditions such as drought, salinity, heat and cold individually cause extensive losses to agricultural production globally, which further aggravates under field conditions due to the simultaneous exposure of plants to more than one abiotic stress factors^9^. It has also been revealed that simultaneous occurrence of more than one stress leads to huge complexity in plant responses, as the responses and adaptation to such combination of stresses are mostly influenced by diverse and at times by antagonistic signaling pathways that may interact and impede each other^6^,^7^. Therefore lately focus of several stress biology research has been directed towards understanding the molecular mechanisms of plant responses to abiotic stresses and their combinations through transcriptomics or functional genomics approaches. qRT-PCR has emerged as a useful technique for validation of transcriptomics data owing to its precision, accuracy, convenience, speed and sensitivity^20^. However accurate normalization of gene expression remains a major criterion for precise qRT-PCR analysis as normalization helps in adjusting variations introduced at various steps of qRT-PCR arising from sample-to-sample variations, variations in RNA integrity, PCR efficiency and cDNA sample loading^22^,^30^. An ideal reference gene thus has constant expression despite of experimental conditions, developmental stages, tissues and cell types^20^,^31^. *Actin, tubulin,18S, glyceraldehyde-3-phosphate dehydrogenase (GAPDH)* and *elongation factor 1a (EF-1a)* have been commonly used as reference genes and their expression is considered to be stable since they are present in all nucleated cell types and are involved in primary metabolism or other cellular processes necessary for cell survival^32^. However there are a number of reports on variable expression of these housekeeping genes in different plant species under different treatments as they could also go through substantial molecular regulations in different environmental conditions and in specific cellular functions^5^,^28^,^30^,^33^,^34^,^35^. Erroneous results and inaccurate gene expression data could be obtained if suitable reference genes are not used^36^. As for example, in tall fescue the expression of *FaWRKY* was found to be increased when *SAND* and *TUB* or *GAPDH* and *TUB* were used for normalization in salt-treated roots and polyethylene glycol-treated leaves, respectively but fluctuations in expression pattern was observed when *EF-1α* was used for gene quantification^34^. Therefore it is necessary to identify and utilize suitable internal control gene(s) with stable expression in different crop species, biological samples and experimental conditions for reliable transcript measurements through qRT-PCR^20^,^37^. In fact more than one study has also been carried out in a single crop to evaluate different control genes for optimal normalization in different experimental conditions eg. rice^25^,^38^,^39^, cucumber^32^,^40^, coffee^41^,^42^, soybean^36^,^43^,^44^, sugarcane^45^,^46^ and pearl millet^47^,^48^.

Pearl millet is one of the most important small-grained annual cereal crops grown in arid and semi-arid regions of Africa and Asia which are usually characterized by scanty rainfall, poor soil fertility and high temperature^16^. It is thus enforced to thrive in such harsh environments, where co- occurrence of one or more than one stress factors is a common phenomenon. Despite these it is well adapted to such adverse environmental conditions and known for its excellent nutritional benefits, and hence is attracting plant researchers to examine its cellular and molecular mechanisms of stress tolerance through transcriptomics or functional genomics approaches. A good understanding of the molecular mechanisms of abiotic stress responses in crop plants will not only help in improving yield but also aid to breeding and transgenic approaches^2^. This study thus investigates 10 potential housekeeping genes for the gene expression analysis in a total of 204 pearl millet samples out of which four are traditional (*ACT*, *EF-1α*, *eIF4A*, and *UBC-E2)* and six new (*GlutR*, *HSP90*, *MDH*, *PP2A, RPL20*, and *TIP41*) reference genes under different abiotic stresses and their combinations at early (1 h) and late (24 h) stress durations in parents of a mapping population differing for drought tolerance as compared to previous studies in pearl millet that were performed under individual stress conditions at one time point only where no contrasting genotypes were used for analysis^47^,^48^. Further the best and worst reference gene sets were validated through expression profiling of a *PgAP2-like ERF* gene from pearl millet.

Several statistical algorithms such as geNorm^28^, NormFinder^49^, BestKeeper^50^, Stability Index^37^, ΔCt^51^ and RefFinder^52^ have been developed to assess the expression stability of candidate reference genes for accurate normalization in gene expression studies and it is also assumed that a comparison of different algorithms allows reliable evaluation^32^. geNorm analysis revealed MV < 1.5 for all 10 reference genes under different experimental conditions in this study indicating their potential expression stability^28^,^29^. The comprehensive results obtained on the basis of statistical analysis by geNorm, NormFinder and RefFinder showed consistency in determining the most stable candidate internal control genes in this study. All the three algorithms recommended *EF-1α* and *UBC-E2* as the top two most stable reference genes in the all samples set across all treatments with some variability in their ranking order indicating the accuracy of our experimental findings. *EF-1α* also constituted the most stable reference gene pair with either *UBC-E2* or *PP2A* according to geNorm in the individual abiotic stress and combinations of abiotic stresses samples set, respectively. Our findings are in confirmation of several previous studies where *EF-1α* was also established as the most stable reference gene as for example, wheat under different abiotic and biotic stresses^26^, cucumber under osmotic and salt stress^32^, soybean under drought and salinity stress^36^, sugarcane under drought and salinity stress^45^, poplar under cold stress^53^, and bermuda grass under drought stress^52^. Interestingly out of six new reference genes used in this study, only *PP2A* could make to the top three stable genes. There are several reports where both old and new reference genes are found to be suitable for normalization^26^,^36^,^54^. In fact *PP2A* was also ranked among best five reference gene in pearl millet in an earlier study^47^. Rest five new genes have low stability with *RPL20* ranked as the least stable reference gene by NormFinder and RefFinder in all samples set. geNorm also ranked *RPL20* as the second least stable gene after *GlutR*. However in the developmental tissues *MDH*, *HSP90* and *EF-1α* were the top three most stably expressed reference genes. The ubiquitous expression of *HSP90* and *EF-1α* is not surprising because they were also reported as valid reference genes across various developmental stages in chickpea^29^. *EF-1α* was also reported to be the most stable reference gene in different tissues of pearl millet however *MDH* showed constant expression in different abiotic stresses only according to a previous study^48^. *HSP90* was not included for analysis in earlier two studies on pearl millet but our study showed it to be the second most stable internal control gene after *MDH* across different developmental stages. However *HSP90* was one of the most variably expressed reference genes in individual and multiple stress conditions, thus limiting its use as reference gene under abiotic stress conditions.

Several studies in various plants have shown that inclusion of more than one reference gene would help in more precise normalization of gene transcript for qRT-PCR analysis^28^,^55^. Pairwise variation analysis by the geNorm applet suggests inclusion of one or more gene for accurate normalization when the value of pairwise variation is above the cut off range of 0.15. Both the earlier studies in pearl millet suggested the inclusion of one or more genes for normalization of gene transcripts^47^,^48^. However in this study, across all the four experimental sets the value of pairwise variation was found to be lower than the geNorm pairwise cut off range indicating that inclusion of additional gene for optimal normalization in pearl millet is not necessary against the suggested gene pair similar to some previous reports^28^,^30^,^34^,^56^.

The suitability of the identified two most stable genes namely *EF-1α* and *UBC-E2* and their combination was assessed by quantifying the expression pattern of an *AP2-like ERF* gene, *PgAP2-like ERF* under both individual and multiple abiotic stresses. *PgAP2-like ERF* showed clear amplification profiles when these two genes were used as internal controls for both individual and multiple abiotic stresses. While severe disparities in the expression pattern of *PgAP2-like ERF* were observed when the least stable reference genes *HSP90* and *eIF4A* were used for normalization in qRT-PCR under individual and combination of abiotic stresses, respectively. Our results indicated that *EF-1α* and *UBC-E2* either singly or in combination are suitable for transcript normalization in pearl millet subjected to different abiotic stresses. Similar kind of study was also carried out in tall fescue^34^. Therefore we recommend the use of *EF-1α* and *UBC-E2* as reference genes for diverse experimental conditions including individual or multiple abiotic stresses, hormone stimulus and developmental tissues in different pearl millet genotypes. It is unlikely that the reference genes other than those identified in this study, may act as better candidates for transcript normalization under various experimental conditions in pearl millet. To the best of our knowledge this is the first report on identification and validation of suitable candidate reference genes for accurate transcript normalization for gene expression studies using qRT-PCR in any crop under various individual abiotic stresses, hormone stimulus and their combinations at early and late durations of stress in contrasting genotypes. The identified reference genes would thus facilitate accurate and consistent qRT-PCR gene expression data analysis over a broad range of developmental tissue samples and multiple abiotic stress conditions in pearl millet and related bioenergy crops for functional genomic studies.

## Methods

### Plant materials and stress treatments

Seeds of two pearl millet cultivars namely, PRLT2/89-33 (drought tolerant) and H77/833-2 (drought sensitive) were kindly provided by Dr. Rakesh K. Srivastava, International Crops Research Institute for Semi-Arid Tropics (ICRISAT), Patancheru, India. Seeds were sown in composite soil (peat : vermiculite : sand mixture, 2:2:1) in glass house at 32 ± 2 °C day/28 ± 2 °C night with relative humidity oscillating between 40–70% and natural sunlight at National Phytotron Facility (IARI), New Delhi, India. Plants were supplemented with water and 1/3^rd^ strength Hoagland solution on alternate days. After two weeks of germination, pearl millet seedlings of each genotype were used for stress treatment. They were precultured for 24 h in one-third strength Hoagland’s solution^4^,^57^. The seedlings were then divided into 14 groups with each group containing 6 seedlings, out of which 13 groups were subjected to individual or multiple abiotic stresses or ABA treatment by transferring the seedlings in the following solutions: drought, solutions containing 20% PEG 6000; salinity, saline solutions containing 250 mM NaCl; heat, solutions containing 1/3^rd^ strength Hoagland solution at 42 °C in a growth chamber; cold, solutions containing 1/3^rd^ strength Hoagland solution at 4 °C in a growth chamber; ABA, solutions containing 100 μM ABA; and in 8 different combinations of stresses including drought + salt, solutions containing 20% PEG 6000 and 250 mM NaCl; drought + heat, solutions containing 20% PEG 6000 exposed to 42 °C in a growth chamber; drought + ABA, solutions containing 20% PEG 6000 and 100 μM ABA; salt + ABA, solutions containing 250 mM NaCl and 100 μM ABA; heat + ABA, solutions containing 100 μM ABA exposed to 42 °C in a growth chamber; cold + ABA, solutions containing 100 μM ABA exposed to 4 °C in a growth chamber; drought + salt + ABA, solutions containing 20% PEG 6000, 250 mM NaCl and 100 μM ABA; and drought + salt + heat + ABA, solutions containing 20% PEG 6000, 250 mM NaCl and 100 μM ABA exposed to 42 °C for 1 h (early) and 24 h (late) according to previous studies^4^,^45^,^58^. Unstressed seedlings were maintained as control and were cultured in the same way as those subjected to stress treatments but without the addition of PEG, NaCl, ABA, or temperature stress. Leaves from a total of 162 samples from 13 different stress treatments at two time points as mentioned above including one untreated control of two genotypes in three biological replicates were carefully harvested and immediately frozen in liquid nitrogen and stored at −80 °C until total RNA isolation.

### Developmental tissue samples

For collection of developmental tissue samples, seeds of both cultivars were sown in separate 9′ pots filled with 5 kg autoclaved composite soil under standard green house conditions as mentioned above. A total of 42 tissue samples including leaf, stem, stem sheath, root, panicle at booting stage and grain filling stages, and seeds in three biological replicates from both genotypes were collected at different developmental stages. Tissue samples of leaf, stem, stem sheath and root were harvested after 30 days of sowing (DAS) from plants grown under normal conditions. Panicle at booting stage and grain filling stages were collected at 40DAS and 55DAS, respectively. Fully mature seeds were harvested at 75DAS.

### Sequence retrieval and primer designing

Sequence information of 10 candidate internal control genes were obtained either from pearl millet expressed sequence tag (EST) database at National Centre for Biotechnology (NCBI) or by searching foxtail millet orthologous locus Ids from Phytozome v.9.0. Sequences of 8 reference genes namely, *ACT*, *EF-1α*, *eIF4A*, *GlutR*, *HSP90*, *MDH, RPL20* and *UBC-E2* were retrieved from respective pearl millet EST sequences available at NCBI, while sequences of *PP2A* and *TIP41* were retrieved from foxtail millet Phytozome database (http://phytozome.jgi.doe.gov/pz/portal.html) for subsequent qRT-primer designing and amplification. Primer pairs were designed from the non-conserved regions of the gene or EST sequences using Primer Express 3.0 (Applied Biosystems, USA) with the following parameters: 20 ± 2 mers primer length; 75–150 bp amplicon size; 60 ± 1 °C melting temperature (Tm); 50 ± 5% guanine-cytosine (GC) content including no hairpin structures, self-dimers or self-complementarities at the 3′-end (Supplementary Table 1).

### Total RNA isolation and cDNA synthesis

The total RNA from samples was isolated using RNAiso plus Reagent (TaKaRa, Japan) according to manufacturer’s instructions. RNA concentration of each sample was determined using NanoDrop- 1000 spectrophotometer (NanoDrop Technologies). The OD_260_/OD_280_ nm absorption ratio (1.98–2.01) and OD_260_/OD_230_ (≥2.0), was used to determine the quality and purity of RNA preparations. RNA integrity of each sample was also verified by electrophoresis in 1.2% formaldehyde-agarose gel in 1 × 3-(N-morpholino) propanesulfonic acid (MOPS) buffer prepared using diethyl pyrocarbonate (DEPC) treated water. Approximately 5 μg of total RNA was DNase treated to completely eliminate DNA contamination using RNase free DNase set (Qiagen, Germany). The first-strand cDNA synthesis was carried out using 1 μg of total Dnase free total RNA primed with oligodT primers in a final reaction volume of 20 μl using Maxima H Minus M-MuLV reverse transcriptase (Thermoscientific, USA) following manufacturer’s instructions and stored at −20 °C.

### PCR and qRT-PCR

Specific amplification of all 10 primer pairs from cDNA was checked by PCR. The 20 μl PCR cocktail contained 10× PCR buffer (Sigma, USA), 10 mM dNTPs mix (Thermosceintific, USA), 10 μM each of forward and reverse primers, 1 μl of cDNA, and 0.2 μl of *Taq* polymerase. The PCR amplification program was as follows: an initial denaturation step of 3 min at 94 °C, and 30 cycles of 30 s at 94 °C, 45 sec at 60 °C and 45 sec at 72 °C followed by an extension step of 10 min at 72 °C. PCR products were separated on 2% agarose-Tris Borate EDTA (TBE) gels. Real-time PCR was performed utilizing SYBR Green detection chemistry on a 7500 fast Real- time PCR system v2.0.6 (Applied Biosystems). qRT-PCR reaction mixture contained 1 μl of 5-fold diluted cDNA (equal to 50 ng of initial amount of RNA), 10 μM of each gene-specific forward and reverse primer and 5 μl of 2× Power SYBR Green PCR Master Mix (Applied Biosystems, USA) in a total reaction volume of 10 μl. The qRT-PCR cycling conditions were as follows: an initial denaturation step of 20 s at 50 °C, 10 min at 95 °C, and 40 cycles of 15 s at 95 °C, and 1 min at 60 °C followed by melt curve analysis using default parameters in order to verify the PCR specificity by constant increase in temperature from 60 °C to 90 °C. The PCR efficiency of each primer pair was determined through slope of the amplification curve in the exponential phase, obtained by serial dilution using *E* = 10 (−1/slope)–1 (3.6 ≥ slope ≥ 3.1) by the software itself (Applied Biosystems). Three biological replicates of each sample were used for real-time PCR analysis and three technical replicates were analyzed for each biological sample.

### Data analysis for expression stability of candidate reference genes

Expression stability of the 10 potential internal control genes across all treatments and samples were determined using three statistical algorithms namely geNorm^28^, NormFinder^49^ and RefFinder^52^ http://omictools.com/reffinder-s2857.html) following developer’s instructions. The Cq or Ct values of all reference genes used in the geNorm and NormFinder were converted into relative quantities using the formula 2^−ΔCt^ where ΔCt is the value obtained after subtracting minimum Ct value from each corresponding Ct value (ΔCt = each corresponding Ct value - minimum Ct value). The geNorm program calculated the expression stability value (M) for each candidate reference gene on the basis of the average variations in the expression level of a particular gene against expression levels of all the other control genes. The geNorm software provided in qBasePlus (v2.4) was also used to calculate the pairwise variation (V_n_/V_n+1_) between the two sequential normalization factors (NF_n_ and NF_n+1_) in order to determine the minimum number of reference genes for optimal normalization. NormFinder, another Microsoft Excel based software package, calculated expression stabilities of the candidate reference genes by combining the intra- and inter-group variations in a sample set containing any number of samples organized in any number of groups. The overall recommended inclusive geomean ranking values of the best reference gene(s) were obtained using the ranking results of geNorm and NormFinder algorithms in the RefFinder online tool.

### Validation of reference genes

For the validation of selected reference gene for qRT-PCR analysis under different individual and abiotic stress combinations in pearl millet, the relative transcript accumulation of an objective gene, *Pg AP2-like ERF* was analyzed using the two most stable namely *EF-1α* and *UBC-E2*; and the two most varying reference genes namely *HSP90* and *eIF4A* identified from this study. Sequence of the objective gene *PgAP2-like ERF* was obtained from a pearl millet transcriptome data from another study (data not published). Primer designing and qRT-PCR reactions were carried out as mentioned earlier. *PgAP2-like ERF* was amplified using PgAP2-like ERF_RT_ F: 5′GCAGAAGAGATTGCTGATGA 3′ and PgAP2-like ERF_RT_ R: 5′GAGGGCTTTGAAGAAGAGAG 3′ primer pair with an amplicon length of 101 bp. The amount of transcript accumulated for *PgAP2-like ERF* normalized to the internal control genes used was analyzed using 2^−ΔΔCt^ method^59^. The relative mRNA expression data of *PgAP2-like ERF* were the means of at least three biological replicates with the results presented as the mean fold change values ± SE.

## Additional Information

**How to cite this article**: Shivhare, R. and Lata, C. Selection of suitable reference genes for assessing gene expression in pearl millet under different abiotic stresses and their combinations. *Sci. Rep.* **6**, 23036; doi: 10.1038/srep23036 (2016).

## Supplementary Material

Supplementary Information

## Figures and Tables

**Table 1:** Details of Ct and CV values of each of the selected candidate reference genes tested in pearl millet.

Gene name	Description	Accession no.	Ct ± SD	CV ± SD
ACT	Actin	HM243500	26.4 ± 2.3	5.8 ± 0.3
EF-1α	Elongation factor-1 alpha	EF694165	23.4 ± 2.1	4.5 ± 0.2
eIF4A	Eukaryotic initiation factor 4A	EU856535	23.8 ± 3.1	6.6 ± 0.4
GlutR	Glutaredoxin	GD180652	21.9 ± 2.5	5.3 ± 0.2
HSP 90	Heat shock protein 90	ADP89125	22.8 ± 2.9	9.6 ± 0.9
MDH	Malate dehydrogenase	CD724779	22.2 ± 2.3	15.7 ± 2.6
PP2A	Protein phosphatase 2A	Si017892m	25.6 ± 2.3	5.1 ± 0.2
RPL20	Ribosomal protein L20	KJ490012	21.7 ± 3.4	6.5 ± 0.4
TIP41	Tonoplast intrinsic protein	Si036884m	28.9 ± 2.5	6.7 ± 0.6
UBC-E2	Ubiquitin-conjugating enzyme E2	CD724586	23.2 ± 2.5	5.7 ± 0.3

**Table 2:** Expression levels of different housekeeping genes under study in all four experimental sets.

Gene name	All samples Ct ± SD	Individual stresses Ct ± SD	Multiple stresses Ct ± SD	Developmental tissues Ct ± SD
ACT	26.4 ± 2.3	26.8 ± 2.7	26.4 ± 1.8	24.8 ± 3.7
EF-1α	23.4 ± 2.1	23.7 ± 2.0	23.2 ± 1.6	23.8 ± 4.0
eIF4A	23.8 ± 3.1	23.6 ± 2.5	24.1 ± 2.2	21.2 ± 4.5
GlutR	21.9 ± 2.5	21.5 ± 2.5	21.8 ± 1.9	23.6 ± 4.3
HSP 90	22.8 ± 2.9	23.3 ± 2.7	22.6 ± 2.5	21.8 ± 4.2
MDH	22.2 ± 2.3	21.5 ± 2.0	22.3 ± 1.6	22.9 ± 3.2
PP2A	25.6 ± 2.3	26.0 ± 2.3	25.7 ± 1.7	23.5 ± 3.3
RPL20	21.7 ± 3.4	20.1 ± 2.4	21.5 ± 2.0	26.7 ± 4.3
TIP41	28.9 ± 2.5	28.2 ± 1.8	29.0 ± 2.0	29.4 ± 3.7
UBC-E2	23.2 ± 2.5	23.3 ± 1.9	23.3 ± 1.7	21.1 ± 4.8

**Table 3:** Stability of candidate reference genes under individual stress conditions in pearl millet.

Rank	geNorm		NormFinder		RefFinder	
	Genes	Normalization Value (MV)	Genes	Stability Value (SV)	Genes	Geomean Of Ranking Value
1	EF-1α│UBC-E2	0.286	UBC-E2	0.048	EF-1α	1.19
2			PP2A	0.103	UBC-E2	2.28
3	PP2A	0.317	ACT	0.104	PP2A	3.03
4	ACT	0.363	EF-1α	0.112	TIP41	4.60
5	RPL20	0.455	RPL20	0.140	MDH	4.73
6	HSP90	0.482	HSP90	0.140	ACT	5.42
7	eIF4A	0.492	GlutR	0.154	GlutR	5.6
8	TIP41	0.561	eIF4A	0.167	RPL20	6.47
9	GlutR	0.593	MDH	0.177	eIF4A	9.24
10	MDH	0.603	TIP41	0.197	HSP90	9.46

**Table 4:** Stability of candidate reference genes under multiple stress conditions in pearl millet.

Rank	geNorm		NormFinder		RefFinder	
	Genes	Normalization Value (MV)	Genes	Stability Value (SV)	Genes	Geomean Of Ranking Value
1	EF-1α│PP2A	0.335	PP2A	0.097	EF-1α	1.19
2			EF-1α	0.159	PP2A	3.72
3	UBC-E2	0.419	UBC-E2	0.161	MDH	5.00
4	MDH	0.438	MDH	0.168	TIP41	6.00
5	TIP41	0.445	HSP90	0.183	GlutR	7.45
6	GlutR	0.517	eIF4A	0.194	ACT	7.74
7	eIF4A	0.579	TIP41	0.201	GlutR	5.6
8	ACT	0.597	GlutR	0.215	RPL20	8.74
9	RPL20	0.606	ACT	0.242	HSP90	10.00
10	HSP90	0.677	RPL20	0.244	eIF4A	11.24

**Table 5:** Stability of candidate reference genes in the developmental tissues of pearl millet.

Rank	geNorm		NormFinder		RefFinder	
	Genes	Normalization Value (MV)	Genes	Stability Value (SV)	Genes	Geomean Of Ranking Value
1	EF-1α│MDH	0.094	MDH	0.032	MDH	1.57
2			HSP90	0.034	HSP90	2.45
3	HSP90	0.151	EF-1α	0.053	EF-1α	2.82
4	eIF4A	0.187	UBC-E2	0.059	ACT	4.16
5	PP2A	0.261	eIF4A	0.060	PP2A	4.30
6	UBC-E2	0.271	ACT	0.074	eIF4A	4.76
7	ACT	0.294	RPL20	0.080	GlutR	5.18
8	GlutR	0.376	PP2A	0.082	TIP41	7.77
9	RPL20	0.427	TIP41	0.102	RPL20	8.24
10	TIP41	0.476	GlutR	0.117	UBC-E2	10.01

**Table 6:** Stability of candidate reference genes across all samples tested in pearl millet.

Rank	geNorm		NormFinder		RefFinder	
	Genes	Normalization Value (MV)	Genes	Stability Value (SV)	Genes	Geomean Of Ranking Value
1	PP2A│UBC-E2	0.218	EF-1α	0.061	EF-1α	1.00
2			PP2A	0.064	UBC-E2	2.38
3	EF-1α	0.292	UBC-E2	0.089	PP2A	3.00
4	eIF4A	0.293	eIF4A	0.089	MDH	3.13
5	ACT	0.355	ACT	0.110	GlutR	5.00
6	HSP90	0.374	HSP90	0.114	TIP41	6.09
7	MDH	0.462	MDH	0.124	ACT	6.24
8	TIP41	0.485	TIP41	0.132	eIF-4A	8.24
9	RPL20	0.498	GlutR	0.146	HSP90	8.74
10	GlutR	0.516	RPL20	0.148	RPL20	10.00
