# Supplementary material for: Selection of suitable reference genes for assessing gene expression in pearl millet under different abiotic stresses and their combinations
Source: Sci Rep. 2016 Mar 14;6:23036. doi: 10.1038/srep23036 (PMC4789795; doi:10.1038/srep23036)
Supplement: Supplementary Information [file srep23036-s1.pdf]

Supplementary Data

Selection of suitable reference genes for assessing gene expression in pearl millet under different abiotic stresses and their combinations

Radha Shivhare[1ǂ] and Charu Lata[1, 2ǂ*]

[1]CSIR-National Botanical Research Institute, Rana Pratap Marg, Lucknow-226001, India

[2]National Research Centre on Plant Biotechnology, Pusa Campus, New Delhi-110012, India

ǂ: Contributed equally to this study

[*]Address for correspondence

Charu Lata

CSIR-National Botanical Research Institute,

Rana Pratap Marg, Lucknow-226001, India

Phone: +91-522-2297996, Fax: +91-522-2205839

E-mail: charulata@nbri.res.in; charulata14@gmail.com

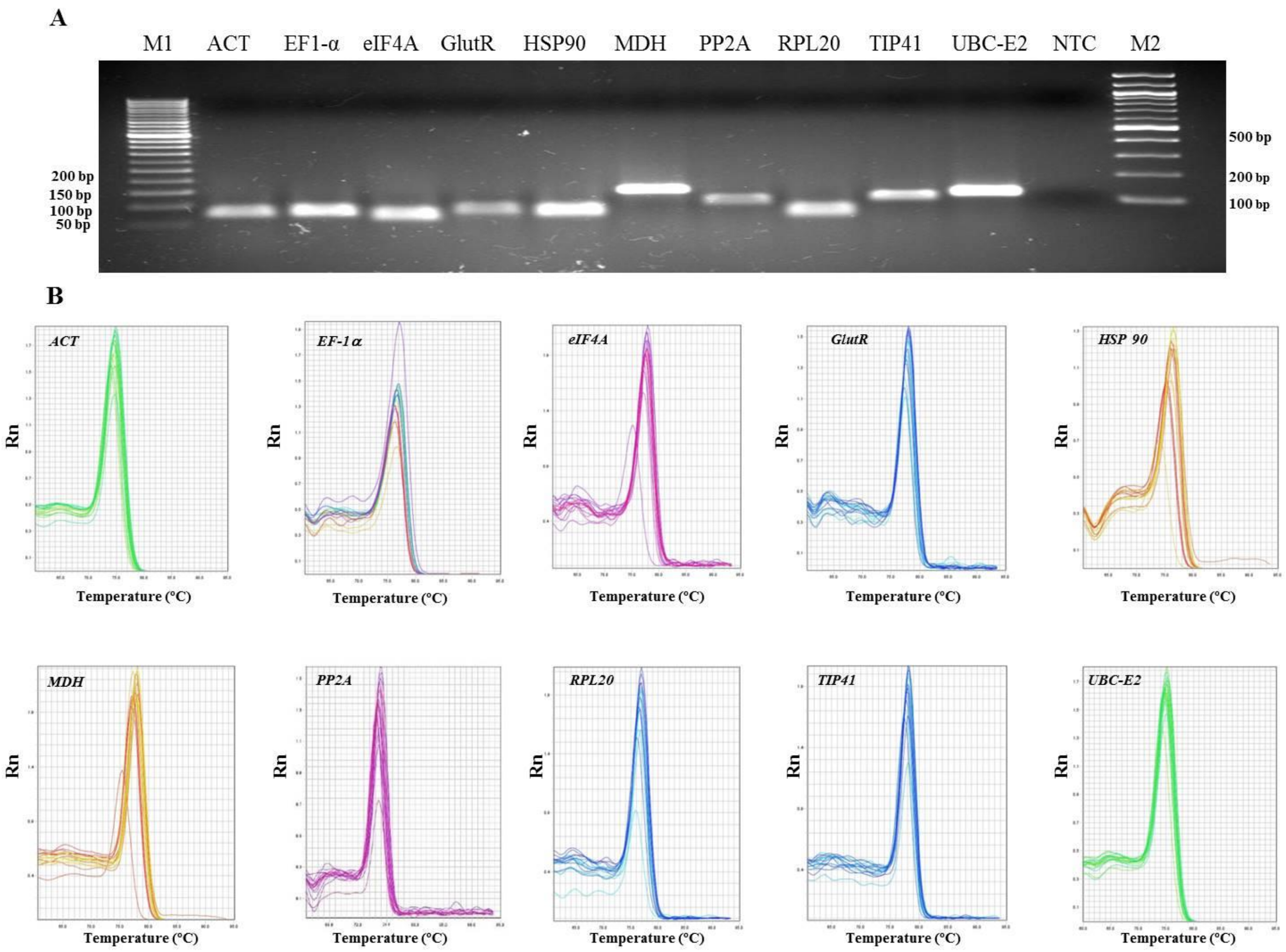

Figure S1. Specificity and confirmation of qRT-PCR amplicon. **(A)**1. 2% formaldehyde-agarose gel showing amplification of single product of the expected size for each reference gene. M1 represent 50 bp and M2 represent 100 bp DNA ladder, respectively. **(B)** Dissociation curve analysis to conform single peak amplification for each primer pair via real-time PCR.

Table S1. Description of primer sequences of candidate reference genes used for qRT-PCR, product size and amplicon characteristics

Genes	Primer pair (5'-3')	Amplicon size (bp)	PCR Efficiency (E)	R^2
ACT	F-AACCCCAAGGCCAATAGAGAGA<br>R-CATACATTGCCGGACATTCAAA	70	0.87	0.94
EF-1α	F- GTTACAACCCAGACAAGATTGC<br>R-TGGACCTCTCAATCGTGTTG	72	0.97	0.97
eIF4A	F-GCGTGCATGTTGTTGTTGGT<br>R-GTGACTGCCTACGCAACATGTC	60	0.93	0.91
GlutR	F-TCCAGCACCCCCCTTATTTT<br>R-TAGCTCGGCGTGAGAGTTGTC	74	1.0	0.92
HSP 90	F-TTGGTCAGTTTGGTGTTGGTTTC<br>R-TGCTTGGTGGTTACGACAACTCT	70	1.0	0.89
MDH	F-CAAGGAAGAAGATGGATGCC<br>R-CGACGCTGGAGTTTCTTTAT	136	0.92	0.98
PP2A	F-TGGTAACATGGCTGCAATTC<br>R-AAATTGAGCCGGACACGA	95	0.93	0.91
RPL20	F-CCCGTGTTGCTGCGTTTAT<br>R-GGGTAGGCAAAAGAGGGATTTT	62	0.89	0.97
TIP41	F-AGTCTGTCTGCGAAAGGATA<br>R-TTCAGCTTCTGGGTCTTCT	104	0.94	0.80
UBC-E2	F-ACCGCCTGACAATCCCTATG<br>R-GGGATAGTCTGGCGGAAAATG	119	1.2	0.93

Table S2. Distribution of the Ct values of each candidate reference genes across the individual stress samples set of pearl millet

**Genes**	**Control**	**Cold 1 h**	**Cold 24 h**	**Heat 1 h**	**Heat 24 h**	**Salt 1 h**	**Salt 24 h**	**Drought 1 h**	**Drought 24 h**	**ABA 1 h**	**ABA 24 h**
ACT	29.3 ± 3.9	28.4 ± 0.7	30.1 ± 0.4	25.8 ± 4.9	26.2 ± 1.2	25.9 ± 1.9	27.0 ± 1.6	25.5 ± 1.5	25.5 ± 1.9	23.4 ± 3.5	28.4 ± 3.7
EF-1α	25.6 ± 1.7	23.6 ± 0.9	22.8 ± 0.5	22.2 ± 2.1	22.9 ± 2.3	22.8 ± 0.6	23.6 ± 1.3	22.6 ± 1.1	23.5 ± 0.5	21.6 ± 1.2	24.5 ± 1.3
eIF4A	25.4 ± 1.2	25.2 ± 1.3	25.7 ± 3.6	22.7 ± 4.5	22.1 ± 3.9	22.8 ± 1.5	23.3 ± 4.0	22.7 ± 1.5	23.8 ± 3.6	21.2 ± 1.6	26.4 ± 4.2
GlutR	23.4 ± 1.4	21.6 ± 1.5	20.1 ± 3.4	23.2 ± 7.8	23.3 ± 3.6	20.3 ± 1.4	21.4 ± 1.5	20.7 ± 0.8	21.2 ± 1.3	19.4 ± 0.5	21.8 ± 0.7
HSP 90	24.1 ± 1.4	24.1 ± 1.5	21.1 ± 1.1	18.3 ± 4.2	21.9 ± 3.2	21.5 ± 2.0	25.3 ± 1.2	22.4 ± 0.2	25.7 ± 0.8	22.1 ± 1.6	27.0 ± 0.7
MDH	22.8 ± 1.2	19.5 ± 1.2	22.1 ± 1.5	21.7 ± 3.6	24.8 ± 1.3	20.3 ± 0.1	22.1 ± 1.1	19.3 ± 1.5	22.4 ± 1.3	19.0 ± 1.2	22.4 ± 0.5
PP2A	28.1 ± 4.2	27.1 ± 3.6	26.6 ± 2.7	27.6 ± 4.1	26.9 ± 1.9	24.3 ± 0.2	26.3 ± 2.4	24.2 ± 0.4	25.6 ± 0.5	22.6 ± 0.6	26.3 ± 1.8
RPL20	22.1 ± 5.4	18.2 ± 0.4	21.0 ± 1.2	20.8 ± 3.4	18.0 ± 4.7	18.8 ± 1.2	21.5 ± 0.9	19.2 ± 0.1	22.0 ± 2.1	19.1 ± 2.3	21.0 ± 0.6
TIP41	31.7 ± 4.5	27.9 ± 4.7	28.1 ± 1.9	29.6 ± 1.5	29.2 ± 0.1	28.4 ± 0.7	28.9 ± 2.3	27.2 ± 0.2	28.8 ± 0.9	26.4 ± 2.7	25.6 ± 1.6
UBC-E2	25.0 ± 2.7	26.5 ± 4.7	25.1 ± 2.5	22.9 ± 3.4	23.3 ± 1.8	22.4 ± 0.6	23.6 ± 1.4	22.0 ± 0.6	23.0 ± 0.7	20.4 ± 1.6	23.0 ± 0.1

Table S3. Distribution of the Ct values of each candidate reference genes across the multiple stress samples set of pearl millet

Genes	Cold +ABA 1 h	Cold +ABA 24 h	Salt +ABA 1 h	Salt +ABA 24 h	Heat +ABA 1 h	Heat +ABA 24 h	Drought +ABA 1 h	Drought +ABA 24 h	Drought +Salt 1 h	Drought +Salt 24 h	Drought +Heat 1 h	Drought +Heat 24 h	Drought + Salt + ABA 1 h	Drought + Salt + ABA 24 h	Drought + Salt + Heat + ABA 1 h	Drought + Salt + Heat + ABA 24 h
ACT	28.3 ± 2.9	26.9 ± 3.1	27.6 ± 0.6	28.5 ± 6.5	26.0 ± 0.3	27.2 ± 3.3	26.8 ± 3.2	25.2 ± 1.5	25.6 ± 1.0	25.3 ± 1.4	26.8 ± 1.5	27.5 ± 0.5	25.6 ± 0.8	24.6 ± 2.6	25.7 ± 0.1	27.4 ± 1.3
EF-1α	24.2 ± 2.6	22.5 ± 2.5	25.4 ± 2.5	23.7 ± 1.4	23.2 ± 2.3	23.5 ± 3.2	22.6 ± 0.2	23.9 ± 1.5	21.6 ± 0.5	23.2 ± 1.6	22..3 ± 1.5	24.7 ± 0.6	21.6 ± 0.9	23.9 ± 1.0	22.9 ± 1.1	22.4 ± 1.4
eIF4A	24.8 ± 0.8	23.4 ± 0.9	27.5 ± 3.2	27.8 ± 4.6	23.2 ± 4.4	24.3 ± 1.6	23.4 ± 1.6	23.9 ± 3.1	22.5 ± 2.6	23.3 ± 3.0	22.9 ± 2.3	24.6 ± 1.6	22.6 ± 2.5	24.2 ± 0.8	22.7 ± 2.9	25.2 ± 0.6
GlutR	22.3 ± 0.5	19.9 ± 0.6	24.2 ± 1.5	22.2 ± 0.5	21.8 ± 4.7	22.1 ± 0.9	21.8 ± 1.7	21.0 ± 2.4	21.6 ± 1.5	21.0 ± 3.4	22.0 ± 2.4	23.2 ± 2.7	20.4 ± 3.0	21.2 ± 1.6	21.7 ± 2.6	22.9 ± 0.9
HSP 90	21.6 ± 1.6	21.3 ± 2.5	24.2 ± 3.5	25.1 ± 2.7	20.6 ± 4.7	20.9 ± 4.7	23.7 ± 0.8	25.5 ± 1.2	22.9 ± 0.7	24.6 ± 1.1	19.6 ± 1.2	21.4 ± 1.1	23.4 ± 0.2	25.3 ± 0.9	19.2 ± 1.6	21.8 ± 1.6
MDH	30.2 ± 4.3	28.5 ± 3.8	30.5 ± 4.5	29.3 ± 2.0	28.1 ± 0.6	29.9 ± 4.3	28.4 ± 0.9	27.4 ± 2.3	28.8 ± 1.5	28.7 ± 0.7	28.5 ± 0.5	30.6 ± 0.7	26.9 ± 0.2	29.0 ± 0.6	28.6 ± 0.1	30.4 ± 1.8
PP2A	26.3 ± 2.7	24.8 ± 2.9	27.2 ± 2.2	25.2 ± 1.6	26.1 ± 2.9	26.4 ± 4.1	24.7 ± 0.2	26.1 ± 0.3	24.2 ± 0.8	24.8 ± 1.8	25.0 ± 1.6	28.4 ± 0.9	24.4 ± 0.3	25.8 ± 0.5	25.2 ± 1.8	26.5 ± 1.3
RPL20	22.0 ± 3.4	19.7 ± 3.0	20.3 ± 2.7	22.3 ± 1.6	22.1 ± 0.1	21.9 ± 2.7	20.8 ± 3.3	22.1 ± 4.7	22.3 ± 0.2	22.0 ± 0.3	21.5 ± 2.7	21.8 ± 0.8	22.3 ± 2.7	23.1 ± 2.3	20.9 ± 2.1	21.7 ± 1.9
TIP41	30.2 ± 4.3	28.5 ± 3.8	30.5 ± 4.5	29.4 ± 2.0	28.1 ± 0.6	29.9 ± 4.3	28.4 ± 0.8	27.4 ± 2.3	28.8 ± 1.6	28.7 ± 0.7	28.5 ± 0.5	30.6 ± 0.7	26.9 ± 0.2	29.0 ± 0.5	28.6 ± 0.1	30.4 ± 1.8
UBC-E2	24.0 ± 2.2	22.2 ± 2.0	25.8 ± 3.5	24.2 ± 2.5	22.8 ± 2.3	24.0 ± 3.4	22.4 ± 0.7	23.9 ± 1.1	22.0 ± 0.4	23.0 ± 0.9	22.6 ± 1.6	24.7 ± 0.2	21.9 ± 0.3	22.2 ± 0.7	22.3 ± 1.5	24.2 ± 0.9

Table S4. Distribution of the Ct values of each candidate reference genes across the developmental tissue samples set of pearl millet

**Genes**	**Leaf**	**Root**	**Stem**	**Stem sheath**	**Booting stage**	**Inflorescence**	**Seeds**
ACT	26.9 ± 0.6	30.0 ± 4.7	26.3 ± 2.1	24.6 ± 3.7	20.2 ± 1.2	21.4 ± 0.8	25.5 ± 2.9
EF-1α	16.0 ± 1.3	26.8 ± 1.2	21.3 ± 0.8	29.0 ± 1.2	21.6 ± 0.8	17.2 ± 0.7	20.1 ± 1.1
eIF4A	29.4 ± 0.9	30.4 ± 2.0	22.5 ± 2.4	26.3 ± 4.8	19.2 ± 0.6	20.3 ± 0.6	20.8 ± 2.4
GlutR	26.8 ± 1.0	31.4 ± 3.9	21.9 ± 1.2	24.7 ± 3.0	19.4 ± 1.8	16.8 ± 0.3	20.7 ± 0.8
HSP 90	28.8 ± 1.8	28.4 ± 1.5	23.1 ± 0.3	23.3 ± 1.6	18.7 ± 1.4	20.1 ± 0.6	24 ± 0 .5
MDH	24.2 ± 0.9	29.4 ± 1.0	21.7 ± 1.6	22.2 ± 0.5	19.4 ± 1.8	16.3 ± 1.0	20.0 ± 1.3
PP2A	32.1 ± 2.2	34.7 ± 2.3	29.3 ± 2.0	27.8 ± 1.7	21.5 ± 1.3	21.1 ± 1.8	26.4 ± 1.7
RPL20	26.6 ± 0.8	28.4 ± 2.0	25.6 ± 3.2	23.3 ± 1.8	20.9 ± 2.1	20.8 ± 1.6	25.8 ± 2.5
TIP41	31.7 ± 7.1	32.6 ± 3.8	30.4 ± 3.5	27.2 ± 3.7	22.8 ± 4.9	23.8 ± 2.6	26.7 ± 3.8
UBC-E2	27.1 ± 0.8	31.5 ± 2.9	22.3 ± 0.2	23.5 ± 1.4	18.8 ± 0.9	18.9 ± 0.1	23.0 ± 0.3

Table S5. Expression stability values and rankings of 10 candidate reference genes calculated using geNorm

Rank	Individual stress		Multiple stress		Tissue		All samples	
	**Gene**	**MV**	**Gene**	**MV**	**Gene**	**MV**	**Gene**	**MV**
1	EF-1α │ UBC-E2	0.286	EF-1α │ PP2A	0.335	EF-1α │ MDH	0.094	PP2A │ UBC-E2	0.218
2								
3	PP2A	0.317	UBC-E2	0.419	HSP90	0.151	EF-1α	0.292
4	ACT	0.363	MDH	0.438	eIF4A	0.187	eIF4A	0.293
5	RPL20	0.455	TIP41	0.445	PP2A	0.261	ACT	0.355
6	HSP90	0.482	GlutR	0.517	UBC-E2	0.271	HSP90	0.374
7	eIF4A	0.492	eIF4A	0.579	ACT	0.294	MDH	0.462
8	TIP41	0.561	ACT	0.597	GlutR	0.376	TIP41	0.485
9	GlutR	0.593	RPL20	0.606	RPL20	0.427	RPL20	0.498
10	MDH	0.603	HSP90	0.677	TIP41	0.476	GlutR	0.516

Table S6. Expression stability values and rankings of 10 candidate reference genes calculated using NormFinder

Rank	Individual stress		Multiple stress		Tissue		All samples	
	**Gene**	**Stability**	**Gene**	**Stability**	**Gene**	**Stability**	**Gene**	**Stability**
1	UBC-E2	0.048	PP2A	0.097	MDH	0.032	EF-1α	0.061
2	PP2A	0.103	EF-1α	0.159	HSP90	0.034	PP2A	0.064
3	ACT	0.104	UBC-E2	0.161	EF-1α	0.053	UBC-E2	0.089
4	EF-1α	0.112	MDH	0.168	UBC-E2	0.059	eIF4A	0.089
5	RPL20	0.140	HSP90	0.183	eIF4A	0.060	ACT	0.110
6	HSP90	0.140	eIF4A	0.194	ACT	0.074	HSP90	0.114
7	GlutR	0.154	TIP41	0.201	RPL20	0.080	MDH	0.124
8	eIF4A	0.167	GlutR	0.215	PP2A	0.082	TIP41	0.132
9	MDH	0.177	ACT	0.242	TIP41	0.102	GlutR	0.146
10	TIP41	0.197	RPL20	0.244	GlutR	0.117	RPL20	0.148

Table S7. Expression stability values and rankings of 10 candidate reference genes calculated using RefFinder

Rank	Individual stress		Multiple stress		Tissue		All samples	
	**Gene**	**Geomean of Ranking Value**	**Gene**	**Geomean of Ranking Value**	**Gene**	**Geomean of Ranking Value**	**Gene**	**Geomean of Ranking Value**
1	EF-1α	1.19	EF-1α	1.19	MDH	1.57	EF-1α	1.00
2	UBC-E2	2.28	UBC-E2	2.06	HSP90	2.45	UBC-E2	2.38
3	PP2A	3.03	PP2A	3.72	EF-1α	2.82	PP2A	3.00
4	TIP41	4.60	MDH	5.00	ACT	4.16	MDH	3.13
5	MDH	4.73	TIP41	6.00	PP2A	4.30	GlutR	5.00
6	ACT	5.42	GlutR	7.45	eIF4A	4.76	TIP41	6.09
7	GlutR	5.60	ACT	7.74	GlutR	5.18	ACT	6.24
8	RPL20	6.45	RPL20	8.74	TIP41	7.77	eIF4A	8.24
9	eIF4A	9.24	HSP90	10.0	RPL20	8.74	HSP90	8.74
10	HSP90	9.46	eIF4A	11.24	UBC-E2	10.01	RPL20	10.0
